# Potassium Chloroaurate-Mediated *In Vitro* Synthesis of Gold Nanoparticles Improved Root Growth by Crosstalk with Sucrose and Nutrient-Dependent Auxin Homeostasis in *Arabidopsis thaliana*

**DOI:** 10.3390/nano12122099

**Published:** 2022-06-18

**Authors:** Sandeep Yadav, Poli Yugandhar, Hemasundar Alavilli, Ramesh Raliya, Archita Singh, Shivendra V. Sahi, Ananda K. Sarkar, Ajay Jain

**Affiliations:** 1 National Institute of Plant Genome Research, Aruna Asaf Ali Marg, New Delhi 110067, India; sandeep18887@gmail.com (S.Y.); architasingh0909@gmail.com (A.S.); 2 ICAR-Indian Institute of Rice Research, Hyderabad 500030, India; poliyugandhar@gmail.com; 3 Department of Bioresources Engineering, Sejong University, Seoul 05006, Korea; alavilli.sundar@gmail.com; 4 Aerosol and Air Quality Research Laboratory, Department of Energy, Environmental and Chemical Engineering, Washington University in St. Louis, St. Louis, MO 63130, USA; rameshraliya@gmail.com; 5 Department of Biology, University City Campus, Saint Joseph's University, 600 S. 43rd St., Philadelphia, PA 19104, USA; ssahi@sju.edus.sahi@usciences.edu; 6 School of Life Sciences, Jawaharlal Nehru University, New Delhi 110067, India; anandaksarkar@mail.jnu.ac.in; 7 Amity Institute of Biotechnology, Amity University Rajasthan, Jaipur 303002, India

**Keywords:** Arabidopsis, hydroponic system, KAuCl_4_, gold nanoparticles, sucrose, nutrients, auxin

## Abstract

In a hydroponic system, potassium chloroaurate (KAuCl_4_) triggers the in vitro sucrose (Suc)-dependent formation of gold nanoparticles (AuNPs). AuNPs stimulate the growth of the root system, but their molecular mechanism has not been deciphered. The root system of Arabidopsis (*Arabidopsis thaliana*) exhibits developmental plasticity in response to the availability of various nutrients, Suc, and auxin. Here, we showed the roles of Suc, phosphorus (P), and nitrogen (N) in facilitating a AuNPs-mediated increase in root growth. Furthermore, the recuperating effects of KAuCl_4_ on the natural (IAA) auxin-mediated perturbation of the root system were demonstrated. Arabidopsis seedlings harboring the cell division marker *CycB1;1::CDB-GUS* provided evidence of the restoration efficacy of KAuCl_4_ on the IAA-mediated inhibitory effect on meristematic cell proliferation of the primary and lateral roots. Arabidopsis harboring synthetic auxin *DR5rev::GFP* exhibited a reinstating effect of KAuCl_4_ on IAA-mediated aberration in auxin subcellular localization in the root. KAuCl_4_ also exerted significant and differential recuperating effects on the IAA-mediated altered expression of the genes involved in auxin signaling and biosynthetic pathways in roots. Our results highlight the crosstalk between KAuCl_4_-mediated improved root growth and Suc and nutrient-dependent auxin homeostasis in Arabidopsis.

## 1. Introduction

Nanomaterials with novel properties provide spectacular paradigms for a wide range of applications in biological imaging, diagnostics, therapeutics, and sensors [1]. Among metal-based nanomaterials, gold nanoparticles (AuNPs) are attributed to highly stable electronic and optical properties, tunable size, and tailorable surface properties [2]. However, hazardous chemicals used for the synthesis of AuNPs contribute to environmental toxicity [3]. Therefore, the green synthesis of AuNPs is an economically viable and eco-friendly sustainable alternative [4]. Whole biomass or different parts of plants with a wide variety of bioactive compounds have been employed in the rapid synthesis of AuNPs [5]. However, plant extracts are not suitable for determining the morphophysiological and molecular responses that are triggered during the synthesis of AuNPs. Therefore, the *in planta* synthesis of AuNPs is a viable alternative [6]. X-ray absorption fine structure (EXAFS) and X-ray absorption near edge structure (XANES) determine the oxidation state of an element and the nearest neighboring atom [7]. In *Sesbania drummondii* seedlings, the acquisition of Au^3+^ and its bioreduction to Au^0^ (AuNPs) were demonstrated by EXAFS and XANES, as well as transmission electron microscopy (TEM)-localized AuNPs in the root cells exhibiting a catalytic activity [8]. The function of AuNPs is determined by its geometry, which can be tailored by altering growth conditions during the *in planta* synthesis of AuNPs [6]. 

*Arabidopsis thaliana* (Arabidopsis) was the first plant whose genome was sequenced and is a popular model plant species [9,10,11]. Therefore, Arabidopsis has been used to decipher various morphophysiological and molecular responses that are induced during the *in planta* synthesis of AuNPs [12,13,14,15,16]. Arabidopsis root system architecture (RSA) represents the spatial configuration of primary and lateral roots and plays a pivotal role in the acquisition of nutrients and water [17]. Arabidopsis RSA is extensively modulated when exposed to either an excess or deficiency of the essential nutrients or non-essential toxic heavy metals [18,19,20,21,22,23]. Supplementation of the nutrient medium with sucrose (Suc) is also required for proper root growth [19]. Therefore, Arabidopsis RSA is the epitome of elucidating the effect of various stresses on the growth and development of seedlings. Interestingly, during the *in planta* synthesis of AuNPs in Arabidopsis, low (10 ppm) and higher concentrations (25–100 ppm) of KAuCl_4_ triggered biphasic stimulatory and inhibitory effects on RSA [12,14,24]. 

Auxin plays a key role in the cell proliferation, differentiation, and expansion of roots [25] and exhibits crosstalk with Suc and other nutrients (Jain et al., 2007 [20]; Rai et al., 2015) [22]. However, the role of Suc and different macro (phosphorus [P] and nitrogen [N])- and micro (iron [Fe] and zinc [Zn])-nutrients and their crosstalk with auxin homeostasis during KAuCl_4_-mediated effects on root growth have not been elucidated. Therefore, we first demonstrated the KAuCl_4_-mediated in vitro synthesis of Suc-dependent AuNPs in a hydroponic system by UV–Vis spectroscopy and transmission electron microscopy (TEM). Quantitative morphometric analysis by the ImageJ program revealed that KAuCl_4_ promoted Suc and nutrient-dependent root growth. Furthermore, we showed that auxin IAA caused a perturbation in the morphological and molecular responses of the root system, which KAuCl_4_ could recuperate. The study thus highlighted the pivotal roles of Suc, nutrients, and auxin homeostasis in KAuCl_4_-mediated increased root growth.

## 2. Materials and Methods

### 2.1. Plant Materials and Growth Conditions

Wild-type Arabidopsis (*Arabidopsis thaliana*) ecotype Columbia (Col-0) and the transgenics, *CycB1;*1::CDB-*uid*A [26], *DR5rev::GFP* [27], and *pPIN1:GUS*, *pPIN2:GUS*, *pPIN3:GUS*, *pPIN4:GUS*, and *pPIN7:GUS* [28], were used in this study. Arabidopsis transgenic seeds carrying *DR5:GFP*, *pPIN1:GUS*, *pPIN2:GUS*, *pPIN3:GUS*, *pPIN4:GUS*, and *pPIN7:GUS* were obtained from the Arabidopsis Biological Resource Center [http://abrc.osu.edu, accessed on 31 July 2020]. The element contamination-free autoclavable and reusable hydroponic system was made from a polycarbonate plant culture box with a polypropylene lid (Magenta^TM^ vessel GA-7; W × L × H = 77 mm × 77 mm × 97 mm), polycarbonate sheet (0.030 inches thick) cut into rectangular pieces (4 cm × 8 cm), and notched at the midpoint to fit together into an X-shaped support for a piece (6 cm × 6 cm) of polypropylene mesh (250 µm pore size), as previously described [29]. The pore size of the mesh facilitated the easy penetration of the root system of seedlings through it into the nutrient medium. In a laminar flow hood, surface-sterilized seeds were sown on the autoclaved mesh and placed in a sterile Petri dish at a low (12 seeds around the perimeter of the mesh) and high (100 seeds/mesh) density. The seeds were sown at a low density to minimize the entangling of roots during growth and their subsequent harvesting for the documentation of different RSA traits and reporter gene assays, while they were sown at a high density to collect the bulk root tissue for qRT-PCR analysis. The seeds sown on the mesh (low and high density) and then initially transferred to the hydroponic system containing a modified one-half-strength Murashige and Skoog (MS) medium (pH 7) [30] supplemented with 1.5% (*w*/*v*) sucrose (Suc) (hereinafter referred to as nutrient-rich (NR) medium) for 7 d. Enough NR medium was added to the hydroponic system to ensure that its level remained ~2 mm above the X-shaped polycarbonate support and touched the surface of the mesh with sown seeds. The hydroponic system was maintained under controlled growth conditions (16 h day/8 h night cycle at 22 ± 2 °C and photosynthetically active radiation (PAR) of 80–90 µmol m^−2^ s^−1^ provided by white fluorescent tubes provided). In 7 d, seeds germinated, and their roots penetrated through the mesh into the NR medium. Seedlings growing on the mesh were gently removed from the hydroponic system and washed thrice each with sterile distilled water and then with different nutrient media, in which they were hydroponically grown for a further 7 d. The following nutrient media were used for growing 7 d-old wild-type and/or transgenic seedlings, and their abbreviated names, which they are referred to hereinafter, are indicated in the parenthesis: (i) NR supplemented with different concentrations (0–100 ppm) of KAuCl_4_, (ii) NR supplemented with 10 ppm KAuCl_4_ (NR.KAuCl_4_), (iii) NR deprived of Suc (Suc-), (iv) Suc- supplemented with 10 ppm KAuCl_4_ (Suc-.KAuCl_4_), (v) KH_2_PO_4_ in MS medium [30] replaced with K_2_SO_4_ (P-), (vi) P-supplemented with 10 ppm KAuCl_4_ (P-.KAuCl_4_), (vii) NH_4_NO_3_ and KNO_3_ in MS medium [30] replaced with KCl (N-), (viii) N-supplemented with 10 ppm KAuCl_4_ (N-.KAuCl_4_), (ix) FeSO_4_/Na_2_EDTA removed from MS medium (Fe-), (x) Fe-supplemented with 10 ppm KAuCl_4_ (Fe-.KAuCl_4_), (xi) ZnSO_4_·7H_2_O removed from MS medium (Zn-), (xii) Zn-supplemented with 10 ppm KAuCl_4_ (Zn-.KAuCl_4_), (xiii) NR supplemented with 0.1 µM indole-3-acetic acid (IAA) (NR.IAA), (xiv) NR.IAA supplemented with 10 ppm KAuCl_4_ (NR.IAA.KAuCl_4_), (xv) NR supplemented with 0.1 µM 1-naphthaleneacetic acid (NAA) (NR.NAA), (xvi) NR.NAA supplemented with 10 ppm KAuCl_4_ (NR.NAA.KAuCl_4_), (xvii) NR supplemented with 0.1 µM 2,4-dichlorophenoxyacetic acid (2,4-D) (NR.2,4-D), and (xviii) NR.2,4-D supplemented with 10 ppm KAuCl_4_ (NR.2,4-D.KAuCl_4_).

### 2.2. Quantification of the Morphological Traits

Seedlings (low density) grown on the mesh were gently removed from the hydroponic system and transferred to a Petri dish containing water. Under a stereomicroscope, the shoots and roots were dissected by a sharp scalpel at the shoot–hypocotyl junction. Furthermore, leaves were dissected from the shoot, transferred to an agar (1%, *w*/*v*) Petri dish, and scanned at 600 dpi (HP scanner). Scanned images were then used for documenting the total shoot area by a Java-based ImageJ processing program [http://rsb.info.nih.gov/ij/, accessed on 15 June 2021], as previously described [31]. Individual dissected roots were immediately transferred to a 1.5 mL Eppendorf tube containing ~1 mL of 70% (*v*/*v*) ethanol and stored in a refrigerator at 3–5 °C. This procedure facilitated storing the roots indefinitely till further documentation of the RSA, which is often a laborious and time-consuming process. To reveal the details of RSA, the individual root was gently removed from the Eppendorf tube and transferred to an agar (1%, *w*/*v*) Petri dish. Under a stereomicroscope, primary and first- and higher-order lateral roots were spread gently with a fine camel-hair brush to ensure that they did not overlap. Spread-out roots were scanned, and the scanned images were then used for measuring the length of the primary root and the number and length of the first- and higher-order lateral roots using the ImageJ program [31].

### 2.3. Transmission Electron Microscopy (TEM)

The formation of AuNPs in the solution was analyzed by TEM. To make the grid hydrophilic, a 400-mesh Formvar^®^ carbon-coated copper grid was glow-discharged for 30 s in a Denton DV 502 vacuum evaporator (Moorestown, NJ, USA). The solution was vortexed, sonicated and an aliquot (2 µL) was carefully pipetted onto the grid. The excess aliquot was wicked off with tissue paper after 30 s. TEM micrographs were captured at 120 kV using FEI Tecnai Spirit TEM (Hillsboro, OR, USA). 

### 2.4. UV–Vis Spectroscopy

The absorption spectrum of AuNPs in the medium was recorded using a UV–Vis spectrophotometer.

### 2.5. Reporter Gene Assay

The roots from the transgenic seedlings (low density) were excised as described for quantifying the morphological traits. For histochemical analysis of the GUS activity in *CycB1;1:CDB-GUS*, *pPIN1:GUS*, *pPIN2:GUS*, *pPIN3:GUS*, *pPIN4:GUS*, and *pPIN7:GUS,* the excised roots of the transgenic seedlings were incubated overnight at 37 °C in a GUS reaction buffer (1 mg mL^−1^ 5-bromo-4-chloro-3-indolyl-*β*-D-glucuronic acid, 5 mM each of K_3_Fe[CN]_6_ and K_4_Fe[CN]_3_H_2_O in 100 mM sodium phosphate buffer [pH 0]) as described [17]. Stained roots (~10–12) for each of the treatments were cleared with 70% (*v*/*v*) ethanol, and GUS activity in the primary root tip was captured by using a differential interference contrast (DIC) microscopy (Axio Imager 2, Carl Zeiss, Jena, Germany). The green fluorescent protein (GFP) images of the primary root tip of transgenics *DR5:GFP* were captured using Axio Imager 2 (Carl Zeiss) and merged with DIC images by employing ZEN lite 2012 analysis software [www.zeiss.com/microscopy/int/products/microscope-software/zen-lite.html, accessed on 11 August 2021].

### 2.6. qRT-PCR Analysis

Wild-type seeds were hydroponically grown at a high density (100 seeds/mesh), and the roots were excised, frozen in liquid nitrogen, and stored at −80 °C till further use. Total RNA was isolated from the root tissue using RNeasy Plant Mini Kit (Qiagen) and treated with RQ1 RNase-free DNase (Promega). DNase-treated RNA (~1 μg) was then reverse-transcribed using the RevertAid First Strand cDNA Synthesis kit (Thermo Scientific, Waltham, MA, USA). Real-time PCR was performed in triplicate for each sample using SYBR green, gene-specific primers, and *UBQ5* (At3g62250) as an internal control on the 7500 Real-Time PCR System (Applied Biosystems). The relative expression levels of the genes were computed by the 2^−ΔΔCT^ method of relative quantification [32]. Primers used for qRT-PCR are provided (Table S1).

### 2.7. Statistical Analysis

Statistical significance of the difference between mean values was determined using Student’s *t*-test. Different letters on the histograms indicate that means were statistically different at *p* < 0.05. 

## 3. Results and Discussion

### 3.1. Medium Composition Affects the Properties of KAuCl_4_-Mediated In Vitro Synthesis of AuNPs in a Hydroponic System

**Figure 1:**
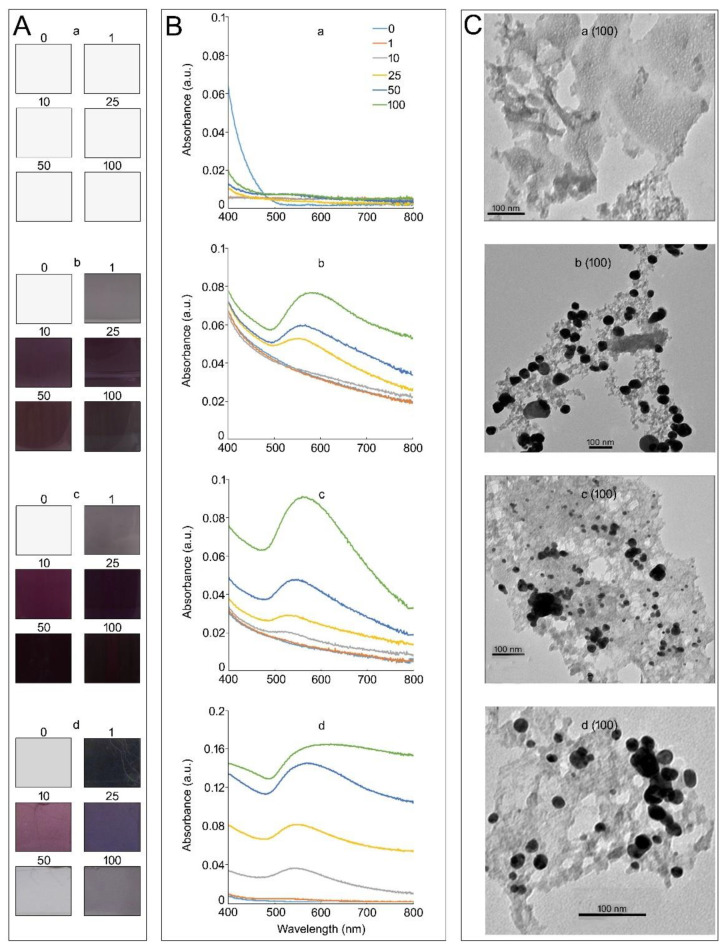
The solution color, UV–Vis spectrum, and TEM images of KAuCl_4_-mediated synthesis of AuNPs in different media. The effects of (**a**) deionized H_2_O, (**b**) one-half-strength MS medium, (**c**) NR comprising one-half-strength MS medium supplemented with 1.5% (*w*/*v*) Suc, and (**d**) wild-type Arabidopsis seedlings grown in NR medium supplemented with 1.5% (*w*/*v*) Suc and different concentrations (0–100 ppm) of KAuCl_4_ for 14 d on (**A**) color and (**B**) UV–Vis spectrum. A shift from colorless to different shades of bluish to bluish–purple and an increase in the absorbance at 530 nm, corresponding to the plasmon absorbance of AuNPs, suggested its formation in the medium in KAuCl_4_ concentration-dependent manner. (**C**) TEM images of AuNPs in different media (**a**–**d**) supplemented with KAuCl_4_ (100 ppm).

Several studies have used a hydroponic system containing a nutrient medium supplemented with KAuCl_4_ for the synthesis of AuNPs in diverse plant species [6,8,12,13,14,15,24].Therefore, the effects of different media (deionized H_2_O, one-half-strength Murashige and Skoog (MS) medium [33], nutrient-rich (NR) medium (MS medium + 1.5% Suc (*w*/*v*)) supplemented with different concentrations (0–100 ppm) of KAuCl_4_, and Arabidopsis seedlings hydroponically grown in NR medium for 14 d were investigated for color changes, the UV–Vis spectrum, and the in vitro formation of AuNPs as revealed by TEM images (Figure 1A–C, first–fourth row). Small monodisperse AuNPs (~30 nm) reflect red light, and as the particle size increases, blue light is reflected. Therefore, changes in the color of the solution are a good indicator of the dosage-dependent KAuCl_4_-mediated synthesis of AuNPs. The concentrated stock solution (10,000 ppm) of KAuCl_4_ (100 mg) prepared in deionized H_2_O (10 mL) has a distinct golden yellow color. No perceptible development of color was observed upon supplementation of deionized H_2_O with different concentrations of KAuCl_4_ (1–100 ppm) (Figure 1A, first row). However, there were perceptible changes in the color of the MS medium from pale blue to bluish–purple upon supplementation with KAuCl_4_ (1 to 100 ppm) (Figure 1A, second row). A similar pattern of color changes was observed when the NR medium was supplemented with different concentrations of KAuCl_4_ (Figure 1A, third row). The results suggest that MS and NR media triggered the formation of AuNPs with different geometries in a KAuCl_4_ concentration-dependent manner. Arabidopsis seedlings were hydroponically grown in NR medium supplemented with different concentrations of KAuCl_4,_ also inducing variable changes in the color of the medium (Figure 1A, fourth row).

The optical properties of AuNPs are sensitive to their sizes, shapes, concentrations, agglomeration states, and refractive indices near their surfaces [34]. Therefore, the surface plasmon resonance of AuNPs could be easily detected in a UV–Vis spectrum as a peak at ~530 nm [35]. Therefore, UV–Vis spectroscopy was used to determine the status of AuNPs in different media supplemented with KAuCl_4_ (0–100 ppm) (Figure 1B). As anticipated, no visible peaks could be detected in deionized H_2_O at any of the KAuCl_4_ concentrations tested (Figure 1B, first row). The result was consistent with no apparent change in the color of deionized H_2_O upon adding different concentrations of KAuCl_4_ (Figure 1A, first row). Interestingly, there were no detectable peaks in the MS medium supplemented with 1 and 10 ppm KAuCl_4_ (Figure 1B, second row), despite changes in the color of these solutions (Figure 1A, second row). The non-detection of peaks could be due to the low amounts of AuNPs formed at these concentrations of KAuCl_4_. A peak was detected at 530 nm when MS medium was supplemented with 25 ppm KAuCl_4,_ and the absorbance value commensurately increased with an increase in the concentration of KAuCl_4_ to 100 ppm (Figure 1B, second row). The addition of 1.5% Suc (*w*/*v*) to the MS medium supplemented with 10 ppm KAuCl_4_ resulted in the detection of a small peak at 530 nm (Figure 1B, third row). Whereas the UV–Vis spectra of MS medium with (Figure 1B, third row) and without Suc (Figure 1B, second row) and supplemented with 25–100 ppm KAuCl_4_ were comparable, albeit with some minor variations in their absorbance values. However, there were significant increases in the absorbance values when wild-type Arabidopsis seedlings were grown in NR medium supplemented with different concentrations (0–100 ppm) of KAuCl_4_ for 14 d (Figure 1B, fourth row) compared with KAuCl_4_ (0–100 ppm)-supplemented NR medium (Figure 1B, third row). UV–Vis spectrum analysis further corroborated the likely effects of exudates from the roots of Arabidopsis on the synthesis of AuNPs in the medium. 

Transmission electron microscopy (TEM) is a commonly used technique for the accurate documentation of the geometry and size distribution of AuNPs [36]. Therefore, TEM images were captured to determine the formation of AuNPs in different media supplemented with KAuCl_4_ (100 ppm) (Figure 1C). AuNPs could not be detected in deionized H_2_O (Figure 1C, first row). The results are consistent with no changes in the color of the solutions and the non-detection of peaks at 530 nm in deionized H_2_O supplemented with KAuCl_4_ (100 ppm) (Figure 1A,B; first row). The distinct formation of AuNPs could be detected in both MS (Figure 1C, second row) and NR (Figure 1C, third row) media, and their sizes were 50 ± 2.6 nm and 10 ± 4.2 nm, respectively. The results suggest that the addition of Suc to MS medium triggered the formation of AuNPs smaller in size. The study revealed the presence of AuNPs in KAuCl_4_ (100 ppm)-supplemented MS and NR media without any plants growing in them. However, when Arabidopsis seedlings were grown for 14 d in KAuCl_4_ (100 ppm)-supplemented NR medium, the sizes of the majority of AuNPs were 12 ± 3.8 nm, of which ~20% were >5 nm (Figure 1C, fourth row). Plant roots continuously secrete an array of chemically diverse compounds into the medium in which they are grown, including sugar alcohols, amino acids, and phenolics [37]. The exudates from the roots of hybrid poplar (*Populus deltoides* × *nigra*, DN34) comprising amino acids, enzymes, mucilage, phenolics, and sugars were presumed to be responsible for a reduction of >90% Au(III) ions into AuNPs (ranging in size from 20 to 40 nm) during growth in a hydroponic solution within 2 d [38]. Therefore, it could be speculated that exudates from the roots of the Arabidopsis seedlings potentially contributed to the formation of AuNPs in the KAuCl_4_ (100 ppm)-supplemented NR medium. AuNPs formed in hydroponics can bind to the carrier proteins and/or organic chemicals and are taken up by the roots through aquaporins or ion channels, transported apoplastically (through intercellular spaces) or symplastically (through plasmodesmata) between cells, and translocated to the shoot along with nutrients and water [38,39].

The temporal effects of Suc in the NR medium on the solution color and UV–Vis spectrum (12 h, 24 h, and 48 h) and the TEM images (48 h) during KAuCl_4_ (100 ppm)-mediated synthesis of AuNPs were also investigated (Figure S1) (see the Supplementary Materials). The changes in the color of the medium from pale blue to bluish–purple (Figure S1A) and increase in absorbance at ~530 nm (Figure S1B) revealed the temporal effect of Suc in NR medium on KAuCl_4_-mediated synthesis of AuNPs (Figure S1C). Suc is a non-reducing sugar, and its progressive hydrolysis into reducing glucose, and fructose (Figure S1D) possibly contributed to the in vitro synthesis of AuNPs. 

### 3.2. KAuCl_4_ Triggers a Dosage-Dependent Augmented Growth Response

**Figure 2:**
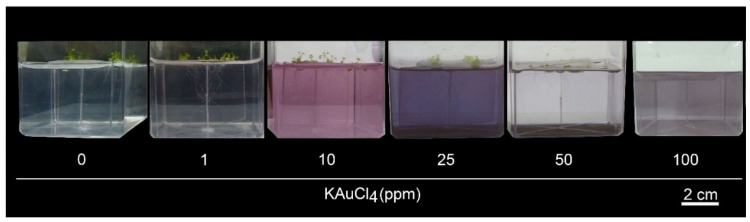
A low dosage of KAuCl_4_ triggers augmented the growth of Arabidopsis seedlings. Wild-type Arabidopsis seedlings were hydroponically grown in a nutrient-rich (NR) medium for 7 d and then transferred to an NR medium supplemented with 0, 1, 10, 25, 50, and 100 ppm KAuCl_4_ and grown for a further 7 d. Dosage-dependent effects of KAuCl_4_ on the development responses of the root and shoot were documented.

**Figure 3:**
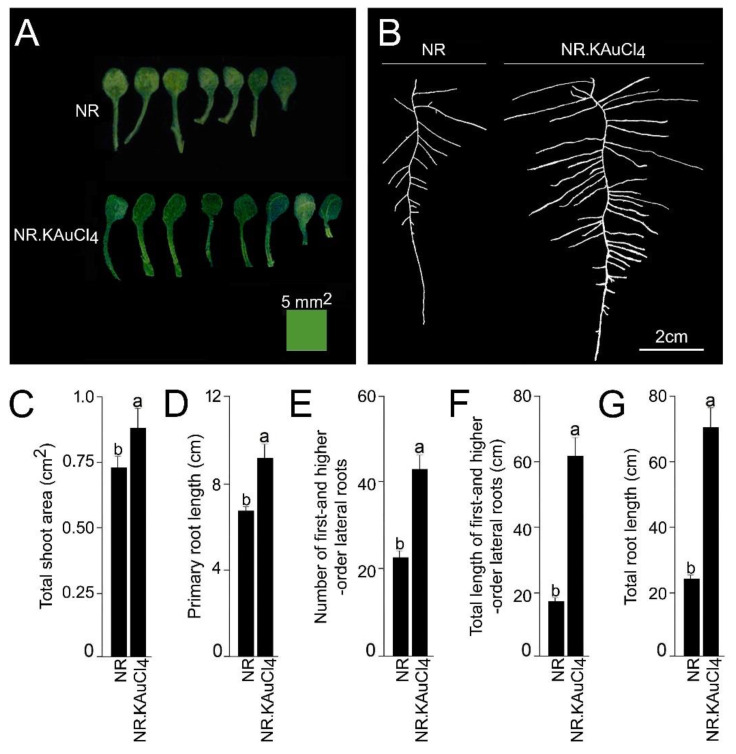
A low dosage of KAuCl_4_ triggered augmented developmental responses of the shoot and root. Wild-type Arabidopsis seedlings were initially grown hydroponically in the NR medium for 7 d and then transferred to the NR medium (control) and NR medium supplemented with 10 ppm KAuCl_4_ (NR.KAuCl_4_) and grown for a further 7 d. The seedlings were removed from the hydroponic system, and then (**A**) shoots and (**B**) roots were separated under the stereomicroscope and spread on an agar plate (1.0%; *w*/*v*) to document their phenotype and quantification of different traits. (**C**–**G**) Data are presented for (**C**) total shoot area, (**D**) primary root length, (**E**) the number of first- and higher-order lateral roots, (**F**) total length of first- and higher-order lateral roots, and (**G**) total root length. Values (**C**–**G**) are means ± SE (*n* = 12) and different letters on the histograms indicate significant differences (*p* < 0.05).

Root system architecture (RSA) comprises the ontogenetically distinct embryonic and post-embryonic development of primary and lateral roots, respectively [17]. RSA exhibits extensive developmental plasticity in response to various environmental cues, including crosstalk effects of various macro- and micro-nutrients, and phytohormone auxin [18,19,21,22]. Therefore, we investigated the dosage-dependent effect of KAuCl_4_ on the growth response of Arabidopsis by growing a wild-type in an element contamination-free and sterile hydroponic system [29] containing a nutrient-rich (NR) medium for 7 d and then transferred to an NR medium supplemented with 0, 1, 10, 25, 50, and 100 ppm KAuCl_4_ and grown for a further 7 d. The dosage-dependent effects of KAuCl_4_ on the growth responses of Arabidopsis seedlings were documented, which varied from no perceptible effect (1 ppm KAuCl_4_), augmented (10 ppm KAuCl_4_), inhibited (25 ppm and 50 ppm KAuCl_4_) and no growth (100 ppm KAuCl_4_) compared with the control (0 ppm KAuCl_4_) (Figure 2). This study revealed a significant effect of KAuCl_4_ on the growth and development of Arabidopsis seedlings in a dosage-dependent manner. Since there was a perceptible augmented growth of Arabidopsis seedlings in the NR medium supplemented with 10 ppm KAuCl_4_ (NR.KAuCl_4_) compared with NR medium (Figure 2), the shoot and root were carefully removed from the hydroponic system, dissected, and separated at the shoot–hypocotyl junction to document their phenotype and quantification of different traits by the ImageJ program (Figure 3A–G). There was an increase in the number and size of the leaflets of NR.KAuCl_4_ compared with NR (Figure 3A), which led to a significant (*p* < 0.05) increase in the total shoot area (Figure 3C). NR.KAuCl_4_ also exhibited robust root growth compared with NR (Figure 3B) due to significant (*p* < 0.05) increases in the primary root length (Figure 3D), number and total length of first- and higher-order lateral roots (Figure 3E,F), which together contributed to a ~3-fold increase in the total root length (Figure 3G). The stimulatory effects of NR.KAuCl_4_ on the growth and development of Arabidopsis seedlings were consistent with earlier studies [12,14]. It was evident from the study that KAuCl_4_ exerted a biphasic dose–response (10 ppm KAuCl_4_: low-dosage-mediated stimulation; 25–100 ppm KAuCl_4_: high-dosage-mediated inhibitory or toxic effect) on the growth and development of the Arabidopsis seedlings. This type of biphasic response to toxic heavy metals is called hormesis, which could be caused by an increase in the production of antioxidants and/or the generation of reactive oxygen species in plants [12,40,41]. Metal nanoparticles at extremely low concentrations (~pg/mL) have also been shown to induce hormetic activation in high-potency homeopathic medicines [42].

### 3.3. KAuCl_4_-Mediated Augmented Growth Response Is Dependent on Suc and Nutrients

**Figure 4:**
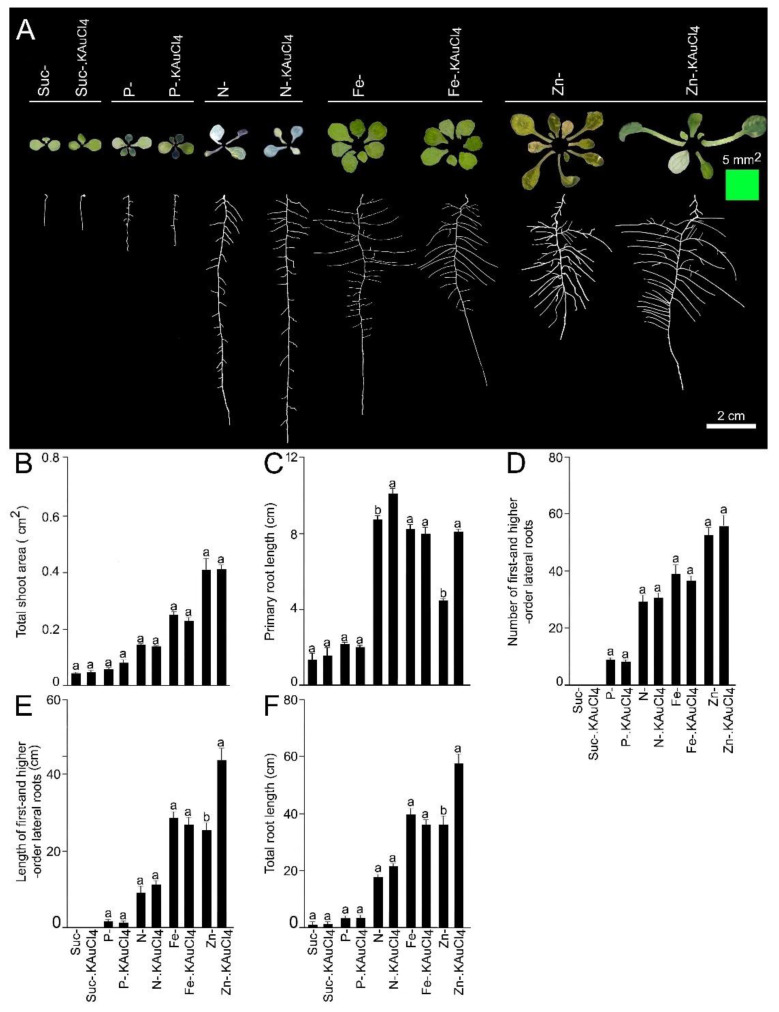
The deficiency of Suc and different essential nutrient elements affected KAuCl_4_-mediated augmented developmental responses of the shoots and roots. Wild-type Arabidopsis seedlings were initially grown hydroponically in NR medium for 7 d and then transferred to an NR medium deprived of Suc (Suc-), Pi (P-), N (N-), Fe (Fe-), and Zn (Zn-), and these media were supplemented with 10 ppm KAuCl_4_ (Suc-.KAuCl_4_, P-.KAuCl_4_, N-.KAuCl_4_, Fe-.KAuCl_4_, and Zn-.KAuCl_4_) for further 7 d. (**A**) The seedlings were removed from the hydroponic system, shoots and roots separated, and spread on an agar plate (1.0%; *w*/*v*) to document their phenotypes. (**B**–**F**) Data are presented for (**B**) total shoot area, (**C**) primary root length, (**D**) the number of first- and higher-order lateral roots, (**E**) total length of first- and higher-order lateral roots, and (**F**) total root length. Values (**B**–**F**) are means ± SE (*n* = 12), and different letters on the histograms indicate significant differences (*p* < 0.05).

Suc and macro- and micro-nutrients play pivotal roles during growth and development of Arabidopsis [18,19,21,22]. Therefore, we investigated the effects of deficiencies of Suc and macronutrients (phosphate [Pi] and nitrogen [N]) and micronutrients (iron [Fe] and zinc [Zn]) on low-dosage KAuCl_4_-mediated augmented growth responses of the shoots and various root traits. Wild-type seedlings were hydroponically grown in an NR medium deprived of Suc (Suc-), Pi (P-), N (N-), Fe (Fe-), and Zn (Zn-), and these media were supplemented with 10 ppm KAuCl_4_ (Suc-.KAuCl_4_, P-.KAuCl_4_, N-.KAuCl_4_, Fe-.KAuCl_4_, and Zn-.KAuCl_4_) for 7 d. The shoot and roots were removed from the hydroponic system, dissected, and separated at the root–hypocotyl junction for documentation of their phenotype and quantification of traits by the ImageJ program (Figure 4A–F). The growth of the shoots and primary roots was highly attenuated under a Suc- condition (Figure 4A–C), and there was no development of first- and higher-order lateral roots (Figure 4D,E), which resulted in a significant (*p* < 0.05) reduction in the total root length (Figure 4F) compared with NR, which contained 1.5% (*w*/*v*) Suc (Figure 3A–G). The results are consistent with an earlier study reporting the key role of Suc in the growth and development of Arabidopsis during growth under controlled growth conditions where white fluorescent tubes provide PAR of 80–90 µmol m^−2^ s^−1^, which is not sufficient for making the plants photosynthetically active, and hence supplementation of the medium is mandatory [19]. A Suc-deprived medium supplemented with KAuCl_4_ (Suc-.KAuCl_4_) could not alleviate the inhibitory effects of Suc- on the developmental responses of the shoot and root, and there were no significant (*p* < 0.05) differences in their values between Suc- and Suc-.KAuCl_4_ (Figure 4A–F). The study revealed that Suc-dependent low-dosage KAuCl_4_-mediated augmented the growth and development of the shoots and roots. 

Among macronutrients, phosphorus (P) is a component of several molecules (ATP, nucleic acids, and phospholipids), playing a key role in signal transduction and various metabolic pathways. Therefore, it is indispensable for the growth and development of plants [43]. Phosphate (Pi) is a bioavailable form of P in soil, and its acquisition by the roots and mobilization to different parts of the plant is mediated by membrane-localized Pi transporters [44]. Pi deficiency (P-) triggered the accumulation of anthocyanin in shoots and significant (*p* < 0.05) reductions in the total shoot area, primary root length, number and length of first- and higher-order lateral roots, and total root length (Figure 4A–F) compared with NR containing P+ (1.25 mM KH_2_PO_4_) (Figure 3A–G). These findings are coherent with earlier studies [19,22]. A P- medium supplemented with KAuCl_4_ (P-.KAuCl_4_) could not recuperate the inhibitory effects of P- on the developmental responses of the shoot and root, and their values were comparable between P- and P-.KAuCl_4_ (Figure 4A–F). Suc plays a key role in various spatiotemporal morphophysiological and molecular adaptive responses of Arabidopsis during growth under different Pi regimes [19]. Thus, this study highlighted the critical role of Pi in KAuCl_4_-mediated augmented growth responses. 

We then investigated whether N availability also influenced the elevated growth responses of Arabidopsis triggered by treatment with low-dosage KAuCl_4_. N is a vital component of chlorophyll, nucleotides, and proteins and is critical for the growth and development of plants [45]. N deficiency (N-) caused leaf chlorosis (insufficient production of chlorophyll), which caused the shoots to become bluish–white (Figure 4A), and there was a significant (*p* < 0.05) reduction in the total shoot area (Figure 4B) compared with NR containing N+ (2.0 mM NH_4_NO_3_ and 1.9 mM KNO_3_) (Figure 3C). Contrary to P-, the primary root length under N- condition was significantly (*p* < 0.05) longer (Figure 4C) than NR (Figure 3D). The results reveal an antagonistic effect of P- and N- on primary root growth. N- also caused a perceptible and significant (*p* < 0.05) increase in the number of first- and higher-order lateral roots (Figure 4D) compared with NR (Figure 3E). The results suggest the stimulatory effect of N- on the growth of primary and lateral roots, which indicates a systemic foraging strategy that augments the soil volume explored by the root system (Figure 4C,D). However, N- triggered significant (*p* < 0.05) reductions in the length of first- and higher-order lateral roots and total root length (Figure 4E,F) compared to NR (Figure 3F,G). The differential effects (inhibitory and stimulatory) of N- observed on different shoot and root traits (Figure 4A–F) were consistent with earlier studies [20,46]. N- medium supplemented with KAuCl_4_ (N-.KAuCl_4_) could not salvage the inhibitory effects of N- on the developmental responses of the shoots, length of first- and higher-order lateral roots, and total root length (Figure 4A,B,E,F). The number of first- and higher-order lateral roots was also comparable between N- and N-.KAuCl_4_ (Figure 4D). Interestingly, primary root length was significantly (*p* < 0.05) longer in N-.KAuCl_4_ compared with N-, which suggested a stimulatory effect of KAuCl_4_ on primary root growth (Figure 4C). 

Furthermore, the effects of micro-nutrient Fe and Zn availability on the low-dosage KAuCl_4_-mediated elevated growth responses of the shoot and root traits of Arabidopsis were investigated (Figure 4A–F). Fe is a key component of various metabolic processes, including chlorophyll biosynthesis, photosynthesis, respiration. It is also a component of Fe-binding sites and heme, involved in a multitude of redox reactions, and is a vital mineral nutrient for almost all organisms [47]. Fe also plays a critical role in Pi-deficiency-mediated adaptive morphophysiological and molecular responses [22,48]. Although Fe deficiency (Fe-) did not exert any significant influence on shoot color, which remained green (Figure 4A), there was a significant reduction (*p* < 0.05) in the total shoot area (Figure 4B) compared with NR containing Fe+ (0.1 mM FeSO_4_·7H_2_O and 0.1 mM EDTA) (Figure 3C). On the contrary, Fe- caused significant (*p* < 0.05) increases in the primary root length, number, and length of first- and higher-order lateral roots, and total root length (Figure 4C–F) compared with NR (Figure 3D–G). The results agree with an earlier study reporting Fe-deficiency-mediated increased primary root length [49]. Compared with Fe-, Fe-.KAuCl_4_ did not cause any significant (*p* < 0.05) increases in total shoot area, primary root length, number, and length of first- and higher-order lateral roots, and total root length (Figure 4A–F). The results further highlight the role of Fe in regulating the augmented growth responses of the seedlings treated with KAuCl_4_. 

After Fe, Zn is the second-most abundant essential transition metal in organisms and acts as a cofactor of many enzymes involved in protein binding, signal transduction, and transcriptional and translational regulation, but it could be toxic when present in excess [50,51]. Zn also exhibits crosstalk with Fe, which is key to adaptive and defense responses during stress mediated by heavy metals in Arabidopsis [51,52]. Zn- caused chlorosis (yellowing of normally green shoots due to a lack of chlorophyll) (Figure 4A) and significant reductions (*p* < 0.05) in the total shoot area and primary root length (Figure 4B,C) compared with NR-containing Zn+ (3 µM ZnSO_4_·7H_2_O) (Figure 3C,D). On the contrary, Zn- triggered significant increases (*p* < 0.05) in the number and length of first- and higher-order lateral roots and total root length (Figure 4D–F) compared with NR (Figure 3E–G). The differential responses of primary and lateral roots to Zn- could be attributed to their distinct ontogeny and were consistent with an earlier study [51]. Although total shoot area and the number of first- and higher-order lateral roots were comparable between Zn- and Zn-.KAuCl_4_ (Figure 4A,B,D), there were significant increases (*p* < 0.05) in the primary root length, length of first- and higher-order lateral roots, and total root length in the latter compared with the former (Figure 4C,E,F). Overall, these results reveal the important and differential roles of Suc and nutrients (P, N, Fe, and Zn) in low-dosage KAuCl_4_ (10 ppm)-mediated augmented growth responses of shoots and roots. 

### 3.4. Differential Efficacy of KAuCl_4_ in Recuperating Natural and Synthetic Auxin-Mediated Modulation in RSA

**Figure 5:**
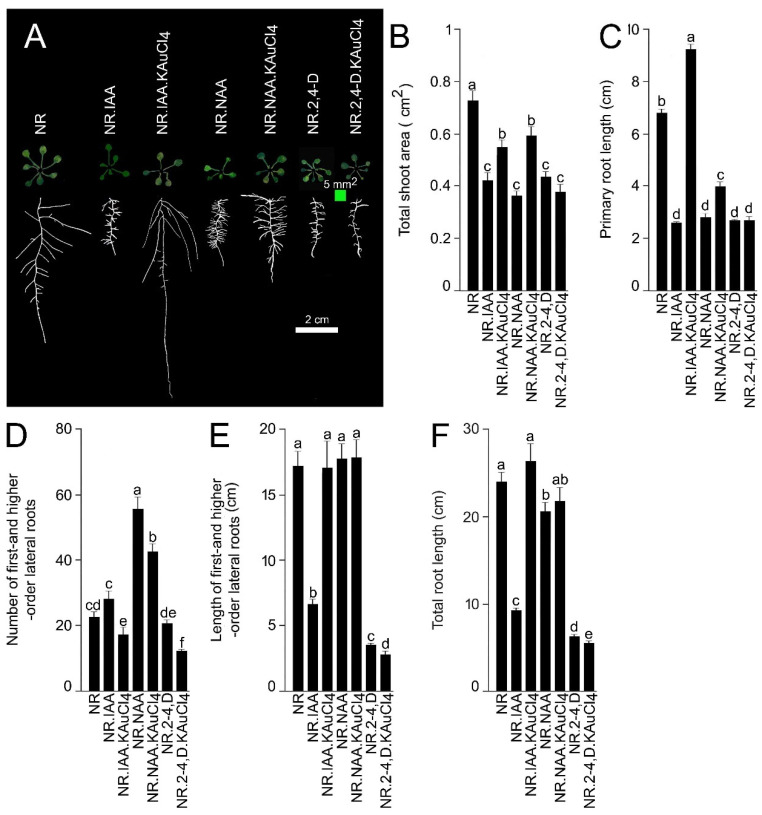
The differential recuperating effects of KAuCl_4_ on the natural and synthetic auxin-mediated perturbation of the developmental responses of the root. Wild-type Arabidopsis seedlings were initially grown hydroponically in the NR medium for 7 d and then transferred to the NR medium supplemented with 0.1 µM each of natural (IAA) and synthetic (NAA and 2,4-D) auxins (NR.IAA, NR.NAA, and NR.2,4-D), and these media were supplemented with 10 ppm KAuCl_4_ (NR.IAA.KAuCl_4_, NR.NAA.KAuCl_4_, and NR.2,4-D.KAuCl_4_) for 7 d. (**A**) The seedlings were harvested, shoots and roots separated, and spread on an agar plate (1.0%; *w*/*v)* to document their phenotype. (**B**–**F**) Data are presented for (**B**) total shoot area, (**C**) primary root length, (**D**) the number of first- and higher-order lateral roots, (**E**) total length of first- and higher-order lateral roots, and (**F**) total root length. Values (**B**–**F**) are means ± SE (*n* = 12) and different letters on the histograms indicate significant differences (*p* < 0.05).

The metabolism, signaling, and transport of phytohormone auxin orchestrates diverse processes of plant growth and development, including apical dominance, root elongation, and responses to phototropic, gravitropic, and various stresses [53]. In Arabidopsis, there is extensive crosstalk between auxin, Suc, and Pi in regulating the developmental responses of the ontogenetically distinct primary and first- and higher-order lateral roots [19]. Although indole-3-acetic acid (IAA) was identified as the key active auxin in most plant species, many other synthetic compounds, including herbicide 2,4-dichlorophenoxyacetic acid (2,4-D) and 1-naphthaleneacetic acid (NAA) revealed auxin-like activities in bioassays [53]. KAuCl_4_ (10 ppm) in NR medium triggered augmented growth in the shoot and root system of Arabidopsis during growth in a hydroponic system (Figure 3).Therefore, to investigate the effects of KAuCl_4_ on the developmental responses of the shoot and root system modulated by the treatment with natural (IAA) and synthetic (NAA and 2,4-D) auxins, wild-type Arabidopsis seedlings were hydroponically grown in the NR medium for 7 d. Seedlings were then transferred to the NR medium supplemented with 0.1 µM each of IAA (NR.IAA), NAA (NR.NAA), and 2,4-D (NR.2,4-D). Furthermore, the media were supplemented with 10 ppm KAuCl_4_ (NR.IAA.KAuCl_4_, NR.NAA.KAuCl_4_, and NR.2,4-D.KAuCl_4_) for 7 d. The seedlings were harvested, shoots and roots separated, and spread on an agar plate (1.0%; *w*/*v*) to document their phenotype and quantification of different traits (Figure 5A–F). There were significant reductions (~43–47%) in the shoot area of the seedlings grown in NR.IAA, NR.NAA and NR.2,4-D compared with NR (Figure 5A,B). The results are coherent with earlier studies reporting the inhibitory effects of IAA, NAA, and 2,4-D on the growth and development of Arabidopsis leaves [54,55,56]. When the seedlings were grown in NR.IAA.KAuCl_4_ and NR.NAA.KAuCl_4_, shoot area increased significantly (~17–32%) compared with NR.IAA, NR.NAA, respectively (Figure 5A,B). The study revealed the efficacy of KAuCl_4_ in partially recuperating the inhibitory effect of IAA and NAA on shoot growth. However, the shoot area of the seedlings grown in NR.2,4-D and NR.2,4-D.KAuCl_4_ was comparable, which suggested the inability of KAuCl_4_ in mitigating the 2,4-D-mediated inhibitory effect on shoot growth and development. Furthermore, we investigated the effects of NR.IAA, NR.NAA and NR.2,4-D on the developmental responses of different root traits (primary root length, the number of first- and higher-order lateral roots, total length of first- and higher-order lateral roots, and total root length) (Figure 5A,C–F). There were significant reductions (~57–60%) in the primary root length of the seedlings grown in NR.IAA, NR.NAA and NR.2,4-D compared with NR (Figure 5A,C). NR.NAA triggered a significant increase (~2.4-fold) in the number of first- and higher-order lateral roots compared with NR, while NR.IAA and NR.2,4-D did not exert any significant influence on this root trait and were comparable with NR (Figure 5A,D). The total length of first- and higher-order lateral roots in NR.IAA and NR.2,4-D significantly reduced by ~60.0% and ~79.0%, respectively, compared with NR, while NR.NAA did not exert any significant influence on this root trait (Figure 5A,E). The total root length was significantly reduced by ~10%, ~60.0%, and ~73.0%, in NR.NAA, NR.IAA, and NR.2,4-D, respectively, compared with NR (Figure 5 A, F). We further investigated the efficacy of NR.IAA.KAuCl_4_, NR.NAA.KAuCl_4_, and NR.2,4-D.KAuCl_4_ in recuperating the inhibitory effects of NR.IAA, NR.NAA and NR.2,4-D on different root traits. Interestingly, the primary root length of the seedlings grown under NR.IAA.KAuCl_4_ was ~39% and ~3.48-fold higher compared with NR and NR.IAA, respectively (Figure 5C). NR.NAA.KAuCl_4_ could recuperate only ~18% primary root length compared with NR.NAA, while NR.2,4-D.KAuCl_4_ did not exhibit any recuperation efficacy and was comparable with NR.2,4-D (Figure 5C). A significant recuperation in the number of first- and higher-order lateral roots was induced by NR.IAA.KAuCl_4_ (~29.0%), NR.NAA.KAuCl_4_ (~24.0%), and NR.2,4-D.KAuCl_4_ (~41.0%) compared with NR.IAA, NR.NAA and NR.2,4-D, respectively (Figure 5D). Interestingly, the total length of first- and higher-order lateral roots and total root length of NR.IAA.KAuCl_4_ exhibited a complete recuperation of NR.IAA-induced inhibitory effects on these traits as evidenced by their values, which were comparable to NR (Figure 5E,F). Overall, this study provides empirical evidence of the efficacy of KAuCl_4_ in mitigating the adverse effects of IAA on different root traits compared with NAA and 2,4-D. Earlier studies also reported the differential diffusion and carrier-mediated influx and efflux rates of IAA, NAA, and 2,4-D and their variable effects on cell division and cell elongation in the cell lines of *Nicotiana tabacum* [57,58]. Since the efficacy of KAuCl_4_ in recuperating the IAA-mediated inhibitory effects on different root traits was relatively more explicit than by NAA and 2,4-D, for the subsequent studies, the role of KAuCl_4_ (10 ppm) in mitigating IAA-mediated perturbation of auxin signal transduction pathway was investigated. 

### 3.5. KAuCl_4_ Recuperates the IAA-Mediated Inhibitory Effect on Primary Root Growth 

**Figure 6:**
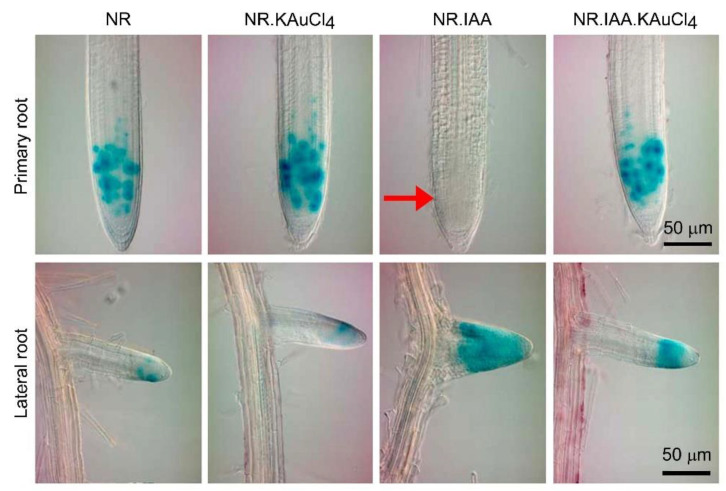
KAuCl_4_ restored the inhibitory effect of IAA on the meristematic cell proliferation of the primary and lateral roots. The transgenic Arabidopsis seedlings harboring the cell division marker *CycB1;*1::CDB-*uid*A reporter gene were initially grown hydroponically in the NR medium for 7 d and then transferred to the NR, NR.KAuCl_4_, NR.IAA, and NR.IAA.KAuCl_4_ media for further 7 d, as described in the legend of Figure 5. Roots were harvested for the histochemical GUS expression analysis of *CycB1;*1::CDB-*uid*A in the primary and lateral roots. The red arrow indicates the effect of NR.IAA on perturbation in the meristematic cell proliferation in the primary root.

The activities of a few stem cells residing at the tips of primary and lateral roots control the overall root system architecture [59]. Auxin governs the root apical meristem (RAM) size by regulating cell division [58]. Since KAuCl_4_ exhibited a significant recuperation of IAA-mediated inhibitory effects on different root traits (Figure 5A–F), its role in mitigating IAA-mediated perturbation in the cell division of primary and lateral root tips was investigated (Figure 6). Eukaryotic cell division is directed by the successive action of cyclin/cyclin-dependent kinase (CYC/CDK) complexes [60]. Mitotic cyclins are under stringent cell-cycle control and accumulate during mitosis, and thus are potent markers for cells undergoing mitosis [61]. In Arabidopsis, mitotic cyclin *CycB1;*1 is expressed only around the G2/M transition of the cell cycle and is transcriptionally regulated [62]. Arabidopsis *CycB1;*1, expressed in the G2/M phase of the cell cycle, was translationally fused to *Escherichia coli uid*A to generate a labile *CycB1;*1::*uid*A reporter for the precise spatio-temporal histochemical analysis of the mitotic activity [63]. Transgenic Arabidopsis expressing *CycB1;*1::*uid*A has been extensively used to demonstrate the Pi-deficiency-mediated progressive loss of meristematic activity in the roots triggering a determinate developmental program that plays a pivotal role in modulating the RSA [19,22]. The cell-cycle-specific ubiquitin-proteasome-mediated rapid degradation of the green fluorescent protein (GFP) was achieved by fusing the cyclin B destruction box (CDB) motif [64]. These studies revealed the enhanced sensitivity of the CDB-fused reporter genes in accurately deciphering the spatio-temporal regulation of gene expression. Transgenic Arabidopsis harboring a translational fusion of chimeric *CycB1;*1::CDB-*uid*A exhibited a tissue-specific post-mitotic expression of *CycB1* [26]. Therefore, transgenic Arabidopsis harboring *CycB1;*1::CDB-*uid*A was used to examine the efficacy of KAuCl_4_ in recuperating IAA-mediated perturbation in the cell division of primary and lateral root tips (Figure 6). Transgenic Arabidopsis seedlings (7-d-old) were hydroponically grown in NR, NR.KAuCl_4_, NR.IAA, and NR.IAA.KAuCl_4_ media for 7 d, and roots were harvested for the histochemical assay of *CycB1;*1::CDB-*uid*A expression in primary and lateral root tips. Histochemical analysis revealed robust expression of *CycB1;*1::CDB-*uid*A in the tips of the primary root of the seedlings grown in NR, NR.KAuCl_4_. The results demonstrate the non-inhibitory effect of KAuCl_4_ on meristematic activity in the primary root tip. On the contrary, there was no expression of *CycB1;*1::CDB-*uid*A in the primary root tip of the seedlings grown in NR.IAA. The red arrow indicates the NR.IAA mediated perturbation of meristematic cell proliferation in the primary root tip. Interestingly, the expression of *CycB1;*1::CDB-*uid*A in the primary root tip of the seedlings grown in NR.IAA.KAuCl_4_ was comparable with NR and NR.KAuCl_4_, highlighting the efficacy of KAuCl_4_ in recuperating the inhibitory effect of IAA on meristematic activity in the primary root tip. The results are coherent with the inhibitory and recuperation effects of IAA and KAuCl_4_, respectively, on the primary root growth (Figure 5A,C). In a study on pea (*Pisum sativum* L.), cobalt (Co) and silver (Ag) ions negated the inhibitory effect induced by ethylene precursor 1-aminocyclopropane-1-carboxylic acid (ACC) but did not mitigate the IAA-mediated inhibition or swelling of the roots [65]. The study suggested that the growth inhibition or swelling of the roots triggered by IAA was not mediated by ethylene and provided evidence of the inhibitory effect of IAA on root growth due to altered auxin homeostasis [65]. Therefore, it is presumed that KAuCl_4_ exerts a significant influence on the auxin-mediated developmental response of the primary root. Unlike the primary root, the expression of *CycB1;*1::CDB-*uid*A in the lateral roots of NR.IAA was not affected and was comparatively more intense compared with NR, NR.KAuCl_4_, and NR.IAA.KAuCl_4_. Primary and lateral roots are embryonic and post-embryonic, respectively, in origin [17], and this could be a plausible explanation for their differential responses to the treatments with IAA and KAuCl_4_. A temporal delay in the loss of meristematic activity in the lateral root tip compared with the primary root tip was also observed in Arabidopsis seedlings deprived of Pi and was attributed to the difference in their ontogeny [22].

### 3.6. KAuCl_4_ Affects Root Growth by Modulating the Components of the Auxin Response Pathways

**Figure 7:**
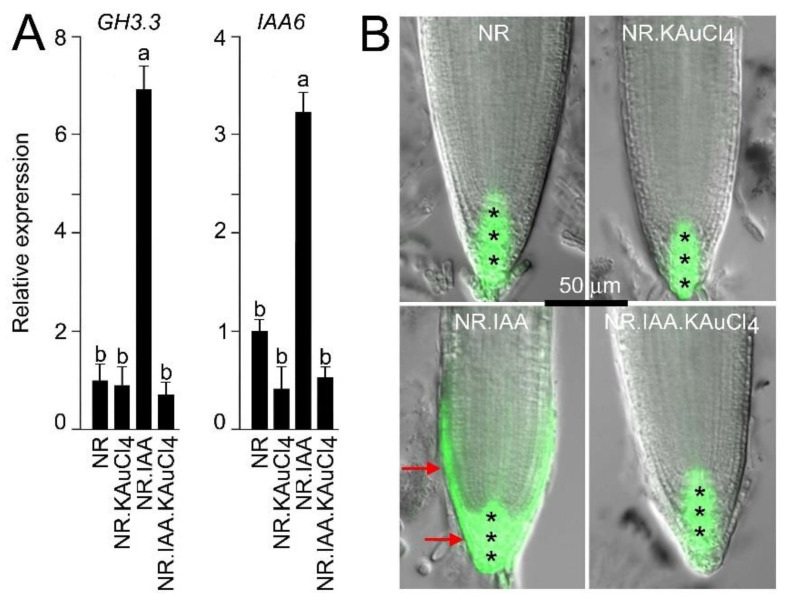
KAuCl_4_ reinstated the IAA-mediated elevated expression of auxin-responsive genes and auxin subcellular localization in the root. Arabidopsis (wild-type and transgenic *DR5rev:GFP*) seedlings were initially grown hydroponically in the NR medium for 7 d and then transferred to NR, NR.KAuCl_4_, NR.IAA, and NR.IAA.KAuCl_4_ media for a further 7 d, as described in the legend to Figure 5. Root tissues of the wild-type and transgenic *DR5rev:GFP* seedlings were harvested for qRT-PCR and fluorescence microscopy, respectively. (**A**) The relative expression levels of *GH3.3* and *IAA6* in the root were determined by qRT-PCR. *ACT2* was used as an internal control. Values are means ± SE (*n* = 6) and different letters on the histograms indicate significant differences (*p* < 0.05). (**B**) Microscopic images of the primary roots showing the effect of NR, NR.KAuCl_4_, NR.IAA, and NR.IAA.KAuCl_4_ on the spatial expression pattern of the transgenic *DR5rev:GFP*. Asterisks (***) indicate the normal expression of *DR5:GFP* in the QC and columella cells. Red arrows indicate the surrounding region of the QC and columella cells where *DR5:GFP* expression was induced upon IAA treatment.

Auxin plays a pivotal role in the growth and development of the root system [66]. The primary root length was significantly reduced during growth in NR.IAA compared with NR, and, interestingly the inhibitory effect of IAA could be circumvented by growing the seedlings in NR.IAA.KAuCl_4_ (Figure 5A,C). This led to an assumption of plausible crosstalk between KAuCl_4_ and auxin sensing and signaling pathways. The distribution patterns and levels of IAA are tightly regulated by synthesis, inactivation by conjugating with sugars or amino acids, and transport [67]. High-NH_4_^+^ stress-mediated inhibition of root growth promoted the conjugation of auxin rather than its inhibition [68]. Several genes from Group II of the *GRETCHEN HAGEN3* (*GH3*) family encode IAA-amido synthetases, which conjugate excess IAA to amino acids to maintain auxin homeostasis, and GH3.3 is one of the GH3 enzymes, which could convert chlorinated IAAs to amino acid conjugates in vitro [69]. The electrophoresis mobility shift assay (EMSA) revealed that the WRINKLED1 (WRI1) transcription factor binds to the promoter of *GH3.3* and plays a pivotal role in maintaining the homeostasis of the root auxin [70]. In addition, Auxin/IAA (Aux/IAA) proteins are the auxin-sensitive transcriptional repressors of the auxin response genes and mediate various developmental and physiological processes [71]. Among the Aux/IAA genes, *IAA6* was shown to play diverse roles, such as controlling the initiation of adventitious roots and mediating drought tolerance by regulating glucosinolate levels [72]. Both *GH3.3* and *IAA6* are the early auxin response genes [73], and thus potent candidates for determining the effects of KAuCl_4_ on their expression. Therefore, qRT-PCR was employed to determine the relative expression levels of *GH3.3* and *IAA6* in the roots of the seedlings grown in NR, NR.KAuCl_4_, NR.IAA, and NR.IAA.KAuCl_4_ media for 7 d (Figure 7A). Although the relative expression levels of *GH3.3* and *IAA6* were comparable under NR and NR.KAuCl_4_, NR.IAA triggered significant increases, which were attenuated and became comparable with NR and NR.KAuCl_4_ upon treatment with NR.IAA.KAuCl_4_. The results suggest that the attenuation of IAA-mediated early auxin response genes by KAuCl_4_. The likely influence of KAuCl_4_ on the IAA-mediated local modulations in auxin subcellular concentrations and localization in the root was assumed. The activity of the synthetic auxin-responsive promoter *DR5* comprising tandem direct repeats of 11 bp, including the auxin-responsive TGTCTC element, has been used for the microscopic visualization of the spatial distribution pattern of auxin [74]. A fluorescent variant *DR5rev::GFP* was constructed as a reliable reporter to monitor auxin response, its dynamics, and cellular levels [27]. Therefore, to investigate the effect of KAuCl_4_ on auxin distribution in the primary root tip, transgenic *DR5rev::GFP* was hydroponically grown in NR, NR.KAuCl_4_, NR.IAA, and NR.IAA.KAuCl_4_ media for 7 d. Fluorescent microscopic images of the primary root tip revealed the effects of NR, NR.KAuCl_4_, NR.IAA, and NR.IAA.KAuCl_4_ on the spatial expression pattern of the *DR5rev:GFP* (Figure 7B). Seedlings treated with NR and NR.KAuCl_4_ showed a normal expression of *DR5rev::GFP* in columella cells and quiescent center (QC). Although the expression of *DR5rev::GFP* in NR.IAA root tip was induced in the surrounding areas of columella cells and QC, it was reinstated in NR.IAA.KAuCl_4_ root tip. The study provided evidence of the effect of KAuCl_4_ on the spatial distribution of auxin in the root tip. 

**Figure 8:**
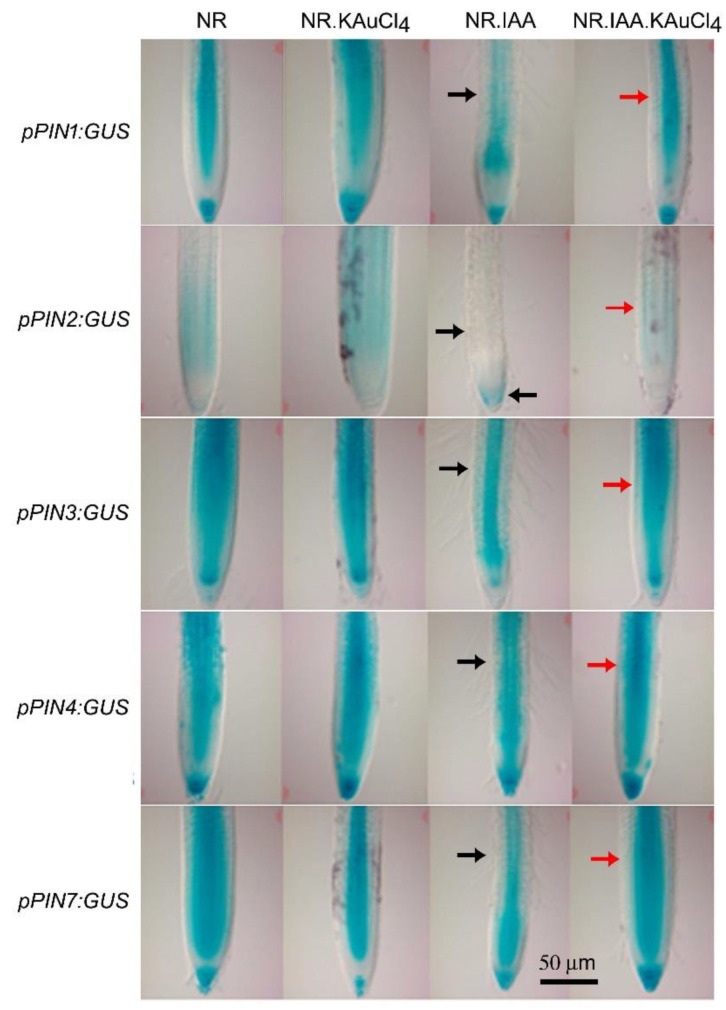
Differential recuperating effects of KAuCl_4_ on IAA-mediated spatial expression patterning of the *PIN* genes in the primary root. The *pPINs:GUS* transgenic seedlings were grown hydroponically in the NR medium for 7 d and then transferred to NR, NR.KAuCl_4_, NR.IAA, and NR.IAA.KAuCl_4_ media for a further 7 d, as described in the legend of Figure 5. Histochemical GUS-stained primary root tip showing the expression of *pPIN1:GUS*, *pPIN2:GUS, pPIN3:GUS, pPIN4:GUS*, and *pPIN7:GUS.* Black arrows indicate the NR.IAA-mediated reduced expression of the *PIN* genes, and red arrows show the effects of NR.IAA.KAuCl_4_ treatment upon the restoration of the spatial expression pattern of these genes.

We further investigated whether KAuCl_4_ exerts any influence on the auxin export carriers that could provide a more in-depth understanding of its observed effects on the auxin fluxes in the root tip (Figure 7B). In Arabidopsis, PIN-FORMED (PIN) are secondary transporter proteins asymmetrically localized within cells; their polarity governs the directionality of intercellular auxin flow and exerts a regulatory influence on an array of diverse developmental responses, including embryogenesis, organogenesis, root and shoot architecture, stem cell maintenance, tissue differentiation, and tropic responses [75]. Among the PIN family members, PIN1-PIN4, PIN6, and PIN7 are PIN auxin export carrier proteins mainly localized at the plasma membrane and facilitate intercellular auxin fluxes [76]. In Arabidopsis root tips, *PINs* exhibited tissue-specific differential expression in vascular tissue (*PIN1*), epidermal and outer cortical cells (*PIN2*), vascular cells and particularly at the basal end of the provascular cells), vascular cells, and largely in the QC and auxin peak region (*PIN4*), and vascular and columella cells (*PIN7*) [28]. *PIN* genes mediate the directional transport of auxin toward the root tip region and their expressions are modulated by both the external and internal cues fluxes [76]. The functional redundancy of PIN proteins and auxin-dependent cross-regulation of *PINs* expression facilitates auxin gradient stabilization, which potentially contributes to the vigor of the adaptive development responses of plants [77]. Therefore, the recuperating effects of KAuCl_4_ on IAA-mediated changes in the spatial expression pattern of *PIN1-4* and *PIN7* reporter lines in the primary root were investigated. Arabidopsis transgenic seedlings were hydroponically grown in NR, NR.KAuCl_4_, NR.IAA, and NR.IAA.KAuCl_4_ media for 7 d, and root tips were excised for histochemical analysis of their GUS activity (Figure 8). Although the expression patterns of *PIN1-4* and *PIN7* were comparable in NR and NR.KAuCl_4_, in NR.IAA, there were variable reductions in their expression patterns with a relatively more profound effect on *pPIN2:GUS*, where it was largely confined to the columella cells of the root tip (indicated by black arrows). However, the modulated and differential GUS activities of *PIN1-4* and *PIN7* in NR.IAA was reinstated in the meristem region of the primary root tip of NR.IAA.KAuCl_4_ (indicated by red arrows). The study thus revealed the recuperating influence of KAuCl_4_ on IAA-mediated differential perturbation of the spatial expression pattern of *PIN1-4* and *PIN7* in the primary root tip.

**Figure 9:**
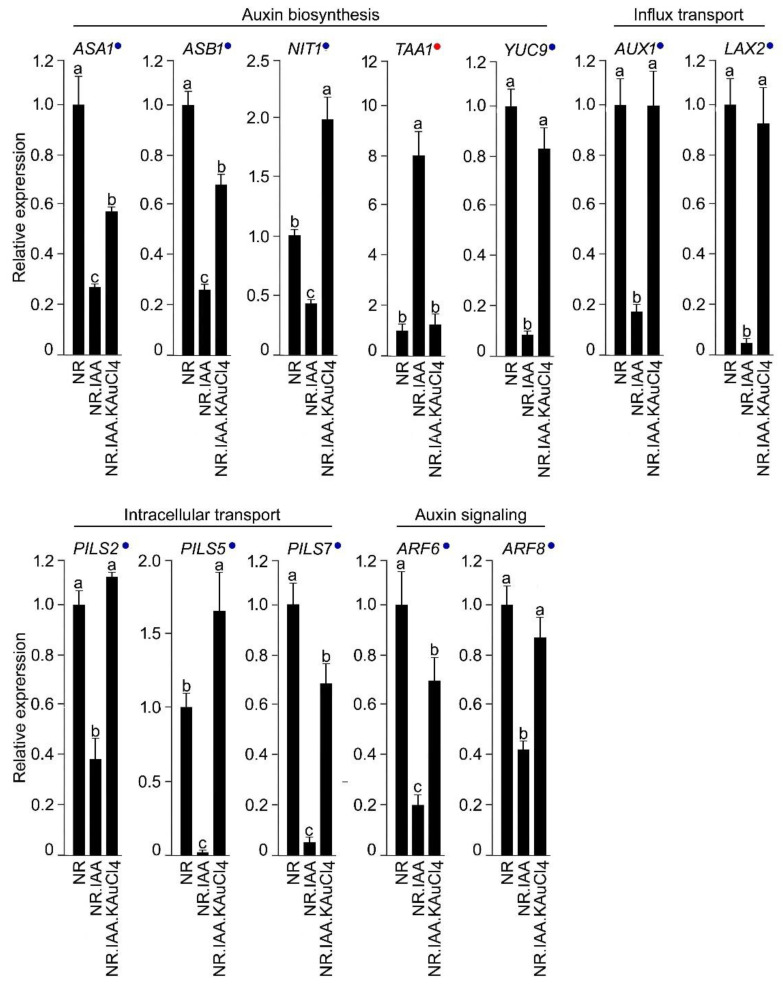
Differential recuperating effects of KAuCl_4_ on IAA-mediated effects on the genes involved in the auxin pathway in the root. Wild-type Arabidopsis seedlings were hydroponically grown in the NR medium for 7 d and then transferred to NR, NR.IAA, and NR.IAA.KAuCl_4_ for a further 7 d, as described in the legend of Figure 5. Roots were harvested, and the relative expression levels of the genes involved in auxin biosynthesis, its influx, intracellular transporters, and signaling were assayed by qRT-PCR. *ACT2* was used as an internal control. Values are means ± SE (*n* = 6) and different letters on the histograms indicate significant differences (*p* < 0.05). Blue and red dots on the histogram indicate the suppression and induction of the genes, respectively, in response to NR.IAA treatment and their subsequent recuperation upon treatment with NR.IAA.KAuCl_4_.

**Figure 10:**
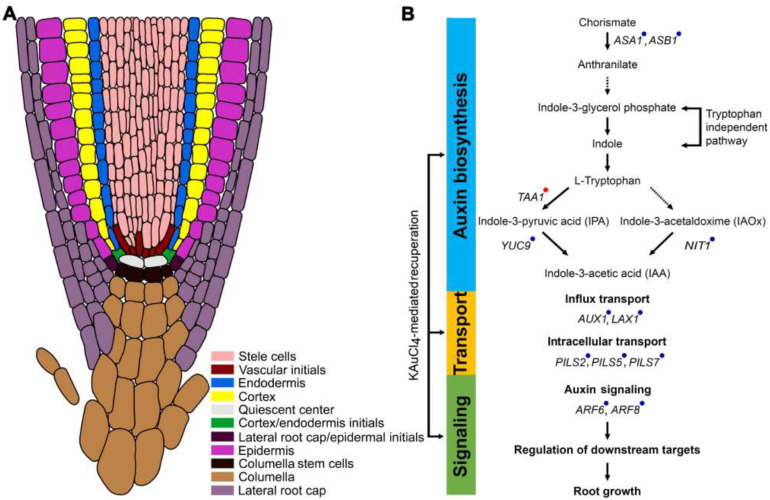
A model depicting the differential effects of KAuCl_4_ on the genes involved in the biosynthesis, transport, and signaling of auxin in the root. (**A**) A schematic diagram of the primary root tip with 11 specific cell types indicated with color codes. *ASA1*, *ASB1*, *NIT1*, *AUX1*, *PILS2*, *PILS7*, *ARF6*, and *ARF8* are expressed in all the cell types. However, *TAA1* (vascular initials, quiescent center, cortex/endodermal initials, lateral root cap/epidermal initials, and columella stem cells), *YUC9* (vascular initials, quiescent center, cortex/endodermal initials, lateral root cap/epidermal initials, columella stem cells, columella, and lateral root cap), *LAX1* (stele cells), and *PILS5* (stele cells, endodermis, cortex, quiescent center, and epidermis) show expressions in only some of the specific cell types. (**B**) Blue and red dots on the genes indicate their suppression and induction, respectively, in response to NR.IAA treatment and their subsequent recuperation upon treatment with NR.IAA.KAuCl_4_. Solid arrows indicate pathways in which the genes, enzymes, or intermediates are known, and dashed arrows indicate pathways that are not well-defined.

Next, we addressed whether KAuCl_4_ exerts any recuperating influence on IAA-mediated modulation in the relative expression of the genes involved in the maintenance of auxin homeostasis. An array of functionally diverse genes is involved in the biosynthesis of auxin (*Anthranilate synthase alpha1* (*ASA1*), *Anthranilate synthase beta1* (*ASB1*), *Nitrilase1* (*NITI*), *Tryptophan aminotransferases of Arabidopsis1* (*TAA1*)*,* and *YUCCA9* (*YUC9*)), its influx (*Auxin1* (*AUX1*) and *Like-Aux2* (*LAX2*)) and intracellular transport (*PIN-Likes* (*PILS2*, *PILS5*, and *PILS7*)), and its signaling (*Auxin Response Factor* (*ARF6* and *ARF8*)), which coordinately play pivotal roles in regulating tissue-specific auxin homeostasis [53,67,75]. Therefore, Arabidopsis wild-type seedlings were hydroponically grown in NR, NR.KAuCl_4_, NR.IAA, and NR.IAA.KAuCl_4_ media for 7 d, roots were harvested, and the relative expression levels of the genes involved in auxin biosynthesis, its influx, intracellular transport, and signaling were assayed by qRT-PCR (Figure 9). The relative expression of all the genes (indicated by blue dots), except *TAAI* (indicated by red dot), significantly reduced in NR.IAA compared with NR. Interestingly, the relative expression of *TAAI* was ~8-fold higher in NR.IAA than NR. However, the relative expressions of many of these genes were reinstated in NR.IAA.KAuCl_4_ and comparable with NR, either completely (*TAAI*, *YUC9*, *AUX1*, *LAX2*, *PILS2*, and *ARF8*) or partially (*ASAI*, *ASB1*, *PILS7*, and *ARF6*). Notably, the relative expression levels of *NIT1* and *PILS5* in NR.IAA.KAuCl_4_ were significantly higher compared with both NR and NR.IAA. This study revealed that KAuCl_4_ recuperated the effects of IAA partially, completely, or with augmentative effects on the relative expression of the functionally diverse genes, which play a pivotal role in the maintenance of auxin homeostasis, which is required for the proper growth and development of plants, including different traits of the root system. A schematic model is presented, highlighting the differential recuperation efficacy of KAuCl_4_ on the cascade of functionally distinct genes, which play significant roles in intricate auxin biosynthetic pathway-mediated root development (Figure 10).

## 4. Conclusions

In the present study, the model plant Arabidopsis was used to investigate the effects of the low-dosage (10 ppm) KAuCl_4_-mediated synthesis of AuNPs on the morphological and molecular responses during growth in a hydroponic system. KAuCl_4_ stimulated the growth of the shoots and root, which was dependent on the availability of Suc and different nutrients, in particular Pi. Since phytohormone auxin plays a vital role in the growth and development of the root system, we then investigated whether there was any perturbation in auxin sensing and signaling cascades during KAuCl_4_-mediated stimulation of the root growth. IAA is a natural and active auxin and caused a significant reduction in the growth of the primary root, which was recuperated upon treatment with KAuCl_4_. The results provide morphological evidence for the effect of KAuCl_4_ on auxin-mediated developmental responses of the root. Furthermore, the use of Arabidopsis transgenics (*CycB1;*1::CDB-*uid*A, *DR5rev::GFP*, *pPIN1:GUS*, *pPIN2:GUS*, *pPIN3:GUS*, *pPIN4:GUS*, and *pPIN7:GUS*) revealed the intricate molecular mechanisms involved in the KAuCl_4_-mediated mitigation of the IAA-induced inhibitory effects on the root growth. Finally, a qRT-PCR analysis highlighted the efficacy of KAuCl_4_ in salvaging the attenuating effects of IAA on cascades of functionally diverse genes involved in the auxin biosynthesis, transport, and signaling. Future studies, employing synchrotron micro-focus X-ray fluorescence (*μ*-XRF) and micro-X-ray absorption near-edge structure (*μ*-XANES) [78] could shed more light on the in situ tissue-specific rates of speciation and bioreduction of KAuCl_4_ (Au^3+^) into AuNPs (Au^0^) in hydroponically grown Arabidopsis under different nutrient regimes. 

## Data Availability

Data are contained within the article.

## Supplementary Materials

The following supporting information can be downloaded at: https://www.mdpi.com/article/10.3390/nano12122099/s1, Figure S1. Effects of Suc in NR medium on the solution color, UV-vis spectrum, and TEM images during KAuCl_4_-mediated synthesis of AuNPs. NR medium containing Suc was supplemented with KAuCl_4_ (100 ppm) and after 12 h, 24 h, and 48 h (A) Color and (B) UV-vis spectrum was documented. (C) TEM images of AuNPs formed in the medium after 48 h. (D) Hydrolysis of non-reducing sucrose into reducing glucose and fructose by the process known as ‘inversion of sugar’. Table S1. List of primers used for qRT-PCR.
