# Supplementary material for: Potassium Chloroaurate-Mediated In Vitro Synthesis of Gold Nanoparticles Improved Root Growth by Crosstalk with Sucrose and Nutrient-Dependent Auxin Homeostasis in Arabidopsis thaliana
Source: Nanomaterials (Basel). 2022 Jun 18;12(12):2099. doi: 10.3390/nano12122099 (PMC9230854; doi:10.3390/nano12122099)
Supplement: Supplementary file 1 [file nanomaterials-12-02099-s001.zip › nanomaterials-1778672-supplementary.pdf]

nanomaterials

MDPI

Supplementary material

Potassium Chloroaurate-Mediated *In Vitro* Synthesis of Gold Nanoparticles Improved Root Growth by Crosstalk with Sucrose and Nutrient-dependent Auxin Homeostasis in *Arabidopsis thaliana*

Sandeep Yadav[1], Poli Yugandhar[2], Hemasundar Alavilli[3], Ramesh Raliya[4], Archita Singh[1], Shivendra V. Sahi[5], Ananda K. Sarkar[6] and Ajay Jain[7,*]

[1]National Institute of Plant Genome Research, Aruna Asaf Ali Marg, New Delhi, India; sandeep18887@gmail.com (S.Y.), architasingh0909@gmail.com (A.S.)
[2]ICAR-Indian Institute of Rice Research, Hyderabad, India; poliyugandhar@gmail.com (P.Y.)
[3]Department of Bioresources Engineering, Sejong University, Seoul, Korea; alavilli.sundar@gmail.com (H.A.)
[4]Aerosol and Air Quality Research Laboratory, Department of Energy, Environmental, and Chemical Engineering, Washington University in St. Louis, St. Louis, Missouri, USA; rameshraliya@wustl.edu (R.R.)
[5]University of the Sciences, 600 South 43rd Street, Philadelphia, PA, USA; s.sahi@usciences.edu (S.V.S.)
[6]School of life sciences, Jawaharlal Nehru University, New Delhi, India; anandaksarkar@mail.jnu.ac.in (A.K.S.)
[7]Amity Institute of Biotechnology, Amity University Rajasthan, Jaipur, India.
* Correspondence: ajain2@jpr.amity.edu

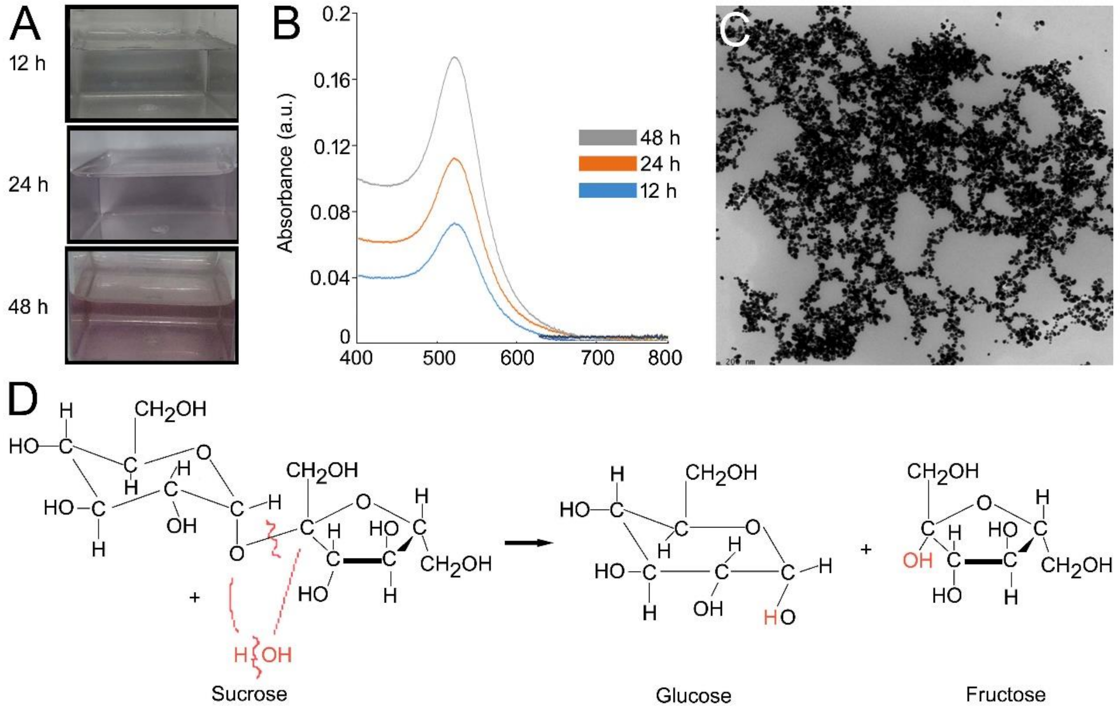

Figure S1. Effects of Suc in NR medium on the solution color, UV-vis spectrum, and TEM images during $KAuCl_4$-mediated synthesis of AuNPs. NR medium containing Suc was supplemented with $KAuCl_4$ (100 ppm) and after 12 h, 24 h, and 48 h A) Color and B) UV-vis spectrum was documented. C) TEM images of AuNPs formed in the medium after 48 h. D) Hydrolysis of non-reducing sucrose into reducing glucose and fructose by the process known as 'inversion of sugar'.

Table S1. List of primers used for qRT-PCR.

Gene	Locus ID	Primers used for qRT-PCR
UBQ5	At3g62250	F: AAGGTTCAGCGTTTGAGGAAG<br>R: GGATCGATCTACCGCTACAACAG
GH3.3	At2g23170	F: CATCACAGAGTTCCTCACAAGCTC<br>R: GGTTGGCATCAACTTCCTTTCACC
IAA6	At1g52830	F: TTCGGCTGTCTTGGCATAGGAG<br>R: TTCACGATCCTCAGCCTCTTGC
ASA1	At5g05730	F: ATGCATATAAGCTCCACGGTGAC<br>R: GTACGTCCCAGCAAGTCAAACC
ASB1	At1g25220	F: ACGAATCCCACAAGAGTTTCCG<br>R: GATTCCGCCATTTCAATCGAAGC
TAA1	At1g70560	F: CTTTCACTCTTCCCAAGTACC<br>R: TGCTCATTACCTTGTGTCTCC
YUC9	At1g04180	F: AGAGATCAGATTGCATAGCGTC<br>R: CGTTGGGTATTCAGGGTAGTG
NIT1	At3g44310	F: GCATTGTACGCCAAAGGCATTGAG<br>R: GAATTGGCAAGCCGACAAGACG
AUX1	At2g38120	F: CAGCTGCGCATCTAACCAAGTG<br>R: GATGAGATAAGCAGTCCAGCTTCC
LAX2	At2g21050	F: TCGGTGGACATGCTGTTACTGTAG<br>R: GCACGTAGAGTGTTGCAAACAGG
PILS2	At1g71090	F: CTCTCTGAAGGACCAAATGA<br>R: CACAATCCCTATTCCCACTAAC
PILS5	At2g17500	F: GAGTCTTGTGGGAGCTTTC<br>R: CAAGTGTAACCGTCTGAGCTA
PILS7	At5g65980	F: CAGTGATTGTGGGAGTGATAAT<br>R: GAGGGTCTGGAGGAAGATAG
ARF6	At1g30330	F: CTCGAAGGCCAATTAGAAGA<br>R: ACGTCGTTCTCTCGGTCAAC
ARF8	At5g37020	F: TTGGGCGTTCATTAGACAT<br>R: GTCATCACCAAGGAGAAGAATA
